# Effect of dance on social physique anxiety and physical self-esteem among adults: a systematic review

**DOI:** 10.3389/fpsyg.2025.1547802

**Published:** 2025-06-04

**Authors:** Xutao Liu, Kim Geok Soh, Yingjie Lu

**Affiliations:** ^1^School of Physical Education, Jiangsu University of Science and Technology, Zhenjiang, China; ^2^Department of Sports Studies, Faculty of Educational Studies, Universiti Putra Malaysia, Serdang, Malaysia; ^3^Department of Music, Faculty of Human Ecology, Universiti Putra Malaysia, Serdang, Malaysia

**Keywords:** psychology, dance, anxiety, self-esteem, mental health, adult

## Abstract

**Background:**

Physical activity has been widely recognized for its positive impact on mental health. Dance, as a form of physical activity, has garnered increasing attention in recent years. Existing literature suggests that dance specifically contributes to enhancing physical wellbeing and promoting emotional development. This systematic review aims to assess the impact of dance on social physique anxiety and physical self-esteem in adults.

**Method:**

A systematic literature search was conducted across multiple academic databases, including Embase, EBSCOhost, Cochrane, Scopus, PubMed, and Web of Science. The data were then systematically reviewed using the PRISMA guidelines. The quality of each study's appraisal was evaluated using the PEDro scale.

**Results:**

Sixteen studies examined the effects of seven types of dances—Zumba, Latin dance, Salsa, Dance Movement Therapy, Aerobic dance, Belly dance, and Colombian Caribbean Folk dance—on adult's social physique anxiety and physical self-esteem. The participants in this review included adults, college students and older adults (ages range from 18 to 76). The quality appraisal scores on the PEDro scale ranged from 3 to 6. Dance interventions were found to significantly enhance physical self-esteem and self-confidence, while concurrently reducing social physique anxiety and negative self-evaluation in an enjoyable manner.

**Conclusion:**

This review indicates that dance positively affected social physique anxiety and physical self-esteem for experimental groups that participated in dance compared to control groups in the reviewed studies. These effects were consistent across all age groups. Moreover, the study demonstrates that female participants in the experimental groups experienced more significant improvements in physical self-esteem and greater reductions in social physique anxiety levels compared to male participants.

**Systematic review registration:**

https://www.crd.york.ac.uk/prospero, identifier: CRD42022315034.

## Background

Approximately 14% of global illnesses can be attributed to mental health issues (Dubreucq et al., 2023). Recent studies have focused on the connection between mental health and physical wellness (Burns et al., 2023), as mental health encompasses both negative attributes such as anxiety and depression (Khanna and Carper, 2022) and positive attributes like self-esteem and a sense of self-worth (Usmani et al., 2023). Promoting mental health is important not only for individuals with existing disorders but for all individuals, as it contributes to overall wellbeing and quality of life (Hart et al., 2022).

Poor mental health can manifest in symptoms such as anxiety, depression, and low self-esteem, many of which are closely related to individuals' body perceptions (Wong et al., 2023). In this review, “mental health” is used as a broad term encompassing both the absence of psychological disorders and the presence of psychological wellbeing. “Psychological wellbeing” specifically refers to individuals' subjective experiences of happiness, self-acceptance, purpose in life, and emotional balance. Individuals may experience anxiety related to their physique or body shape, leading to Social Physique Anxiety (SPA) and low Physical Self-esteem (PSE). SPA refers to individuals' concerns about how their physical appearance is perceived by others in social settings (Zartaloudi et al., 2023). Engaging in physical activity has been proven to have a positive impact on aspects of mental health, such as anxiety and PSE, including improvements in self-confidence and self-perception as subcomponents of PSE, which are frequently affected by body-related concerns (Rogers et al., 2023). One form of physical activity that has gained increasing attention is dance, which has the potential to improve both physical fitness and psychological wellbeing (Hart et al., 2022). Dance has been shown to influence two important psychological constructs, namely SPA and PSE (Soltero et al., 2022). In this review, closely related psychological constructs are distinguished as follows: PSE refers specifically to an individual's evaluation of their own physical appearance and abilities; self-confidence and self-perception, although conceptually related, are regarded as components or broader reflections of self-esteem in physical contexts. For clarity and consistency, the term PSE is employed when referring to body-related self-evaluation, and the interchangeable use of related terms is avoided unless directly cited from original studies.

SPA is characterized by concern over how others may evaluate one's physical appearance (Ng et al., 2023). It can act both as a barrier to exercise, due to concerns about revealing one's physique in a public setting, and as a motivator for participation in exercise to develop a healthier and more desirable body image (Abdollahi et al., 2023). SPA is the fear of negative judgment in social situations (Kroon et al., 2023). PSE, on the other hand, reflects an individual's perception of their body and physical appearance (Alhumaid and Said, 2023). Perceived physical appearance and body image are both essential aspects of PSE, with positive body image playing a particularly central role (Boing et al., 2023). Previous research has shown that physical activity positively influences PSE, especially in adolescents (Marco et al., 2023). Identifying suitable physical exercises to effectively improve SPA and PSE is crucial.

Dance has gained popularity as a physical exercise that can reduce anxiety, boost PSE, and improve mental health (Zhang et al., 2023). Although the impact of dance on health and wellbeing has received less attention compared to other forms of physical activity, studies have shown that it can increase positive feelings such as exhilaration and enthusiasm while decreasing negative emotions like anger, despair, and stress (Lim et al., 2022; Wang, 2023). Dance has been found to have favorable effects on anxiety, sadness, physical function, disability, and memory in adults (Lin et al., 2022; Salihu et al., 2021; Salmons et al., 2022). Various dance styles, including Zumba, Latin, Dance Movement Therapy (DMT), Colombian Caribbean Folk dances, Aerobic dance, Belly dance, and Salsa, have consistently demonstrated positive outcomes such as reduced anxiety, improved mood, and enhanced self-image (Liu et al., 2023). Although DMT is a therapeutic modality rather than a conventional dance genre, it was included in this review because it employs expressive movement and has comparable psychological benefits. Moreover, regular and systematic dance participation has been associated with improved physical health and overall quality of life in older adults (Liu et al., 2021). With their accessibility and, in many cases, relatively low cost compared to other structured physical or psychological interventions, dance programs tend to have lower drop-out rates, making them an effective and sustainable method to enhance mental health (Tomaszewski et al., 2023).

Based on existing literature, women have shown greater participation in dance activities compared to men, with more studies focusing on female participants (Barna et al., 2023; Jiang, 2022). Previous research on SPA and PSE has predominantly focused on adults, especially college students, who are more susceptible to SPA (Zhong et al., 2022). This systematic review aims to summarize the effects of dance on SPA and PSE across different age groups and dance styles. By expanding the study population and considering various dance forms, this review provides substantial theoretical support and promotes the use of dance to improve physical and mental health.

## Methods

### Protocol and registration

The Preferred Reporting Items for Systematic Reviews and Meta-Analyses (PRISMA) recommendations were followed for the data collection, selection, and analysis for this study (Moher et al., 2009). This study was registered with the International Prospective Register of Systematic Reviews (PROSPERO) under ID CRD42022315034.

### Search strategy

The search strategy applied Boolean operators and a strategic combination of keywords across databases. Boolean operators are logical connectors used in search strategies to combine or exclude keywords. Common Boolean operators include “AND”, “OR”, and “NOT,” which help narrow or broaden search results. An in-depth digital search was conducted in July 2024 using several prominent academic databases, including Embase, EBSCOhost, Cochrane, Scopus, PubMed, and Web of Science. The search was conducted using a combination of keywords related to dance interventions, psychological outcomes, and target populations. Additionally, the reference lists of relevant articles and related reviews were manually screened to identify additional eligible studies. All titles were carefully inspected to ensure relevance. Duplicate records were identified and removed using EndNote, and titles were manually screened to avoid repetitions and ensure relevance. Table 1 presents the final set of search terms used across all databases.

**Table 1:** Final search terms.

**Component**	**Search terms**
Population	“adult” OR “adolescent” OR “older adult”
Intervention	“dance” OR “dance movement” OR “Latin dance” OR “Latin” OR “Ballet” OR “Zumba” OR “Modern dance” OR “Classical dance” OR “Jazz dance” OR “Tap dance” OR “Dance Movement Therapy” OR “Salsa dancing” OR “folk dance”
Outcome	“social physique anxiety” OR “anxiety” OR “social anxiety” OR “social physical anxiety” OR “physical activity and anxiety” OR “anxiety about body” OR “nervousness” OR “anxiousness” OR “depress” OR “mood” OR “stress” OR “self-consciousness” OR “distress” OR “loneliness” OR “body image” OR “emotion” OR “anxiety disorders” OR “psychological” OR “psychological distress” AND “physical self-esteem” OR “self-confidence” OR “Confidence” OR “self-esteem” OR “body satisfaction” OR “self-worth” OR “self-image” OR “self-assurance” OR “assertiveness” OR “assuredness” OR “aplomb” OR “cool” OR “poise” OR “self-perception” OR “self-perceptions”

## Inclusion and exclusion criteria

This systematic review used the PICOS (population, intervention, comparison, outcome, study designs) criteria, as shown in Table 2 (Liberati et al., 2009). There were no restrictions regarding sample size, study location, or intervention duration. On this basis, research that met the following criteria were included: (1) A full-text, peer-reviewed study published in English that investigated the effects of at least one type of dance intervention on SPA and/or PSE; (2) Only planned and organized dance intervention; (3) Studies that assessed at least one additional psychological or physical outcome related to body image or physical health (e.g., body satisfaction, emotional wellbeing, or perceived health status); (4) The participants were adults aged 18 years and older.

**Table 2:** PICOS (population, intervention, comparison, outcome, study designs).

**PICOS**	**Detailed inclusion criteria**
Population	Adults aged 18 and above, including university students and older adults
Interventions	Dance-there were no limitations regarding type/style, individual/partnered dances, group/private classes, etc.
Comparisons	Compared to no-intervention or non-dance activity control groups
Outcomes	Social physique anxiety, physical self-esteem
Study designs	RCT or Non-RCT

Studies were discarded in cases where they did not satisfy the mentioned inclusion criteria or met the following exclusion criteria: (1) The articles used the following study designs: cross-sectional, survey, investigation, protocol, and feasibility report without data; (2) Studies were excluded if participants were not generally healthy adults (e.g., those with severe mental illnesses, substance abuse disorders, or cognitive impairments); (3) Articles, meeting abstracts, book sections were not written in English; (4) Articles without an English abstract, containing data deficiencies, or having significant methodological issues (e.g., inadequate sample size, lack of proper controls, or insufficient data reporting).

### Data extraction

After completing the search for relevant articles, data from eligible articles were extracted with a predetermined form, which included: (1) author, title of publication, year of publication; (2) research design or framework; (3) control group and sample size; (4) subject age and gender; (5) intervention characteristics (length, type and frequency); (6) measures index score; and (7) research results. One of the authors abstracted data into a standardized form, while the other validated the data.

### Quality assessment

The Physiotherapy Evidence Database (PEDro) scale (https://pedro.org.au/spanish/learn/pedro-statistics/) is a tool designed to evaluate the methodological quality of experimental designs. It has proven to be valuable when developing systematic reviews due to its high validity and reliability (Moseley et al., 2015).

The PEDro scale is intended to assess a study's four primary methodological components, including randomization, blinding, group comparison, and data analysis. It contains 11 items: Eligibility criteria, Random allocation, Concealed allocation, Baseline comparability, Blind subjects, Blind therapists, Blind assessors, Adequate follow-up, Intention-to-treat analysis, Between-group comparisons, and Point estimates and variability (https://pedro.org.au/wp-content/uploads/PEDro_scale.pdf). However, the first item—“eligibility criteria”—is related to external validity and is not included in the final PEDro score. Thus, although there are 11 items, the maximum score is 10. Two trained independent evaluators using a yes (1 point) or No (0 points) evaluate the quality of trials in the PEDro database, and if there are conflicts, a third evaluator resolves them. The greater the PEDro score, the better the method's quality (Maher et al., 2003). To determine the quality of the method, the following criteria were used: a PEDro score < 5 indicates poor quality, while a score > 5 indicates excellent quality (González-Muñoz et al., 2023).

## Results

### Study selection

**Figure 1:**
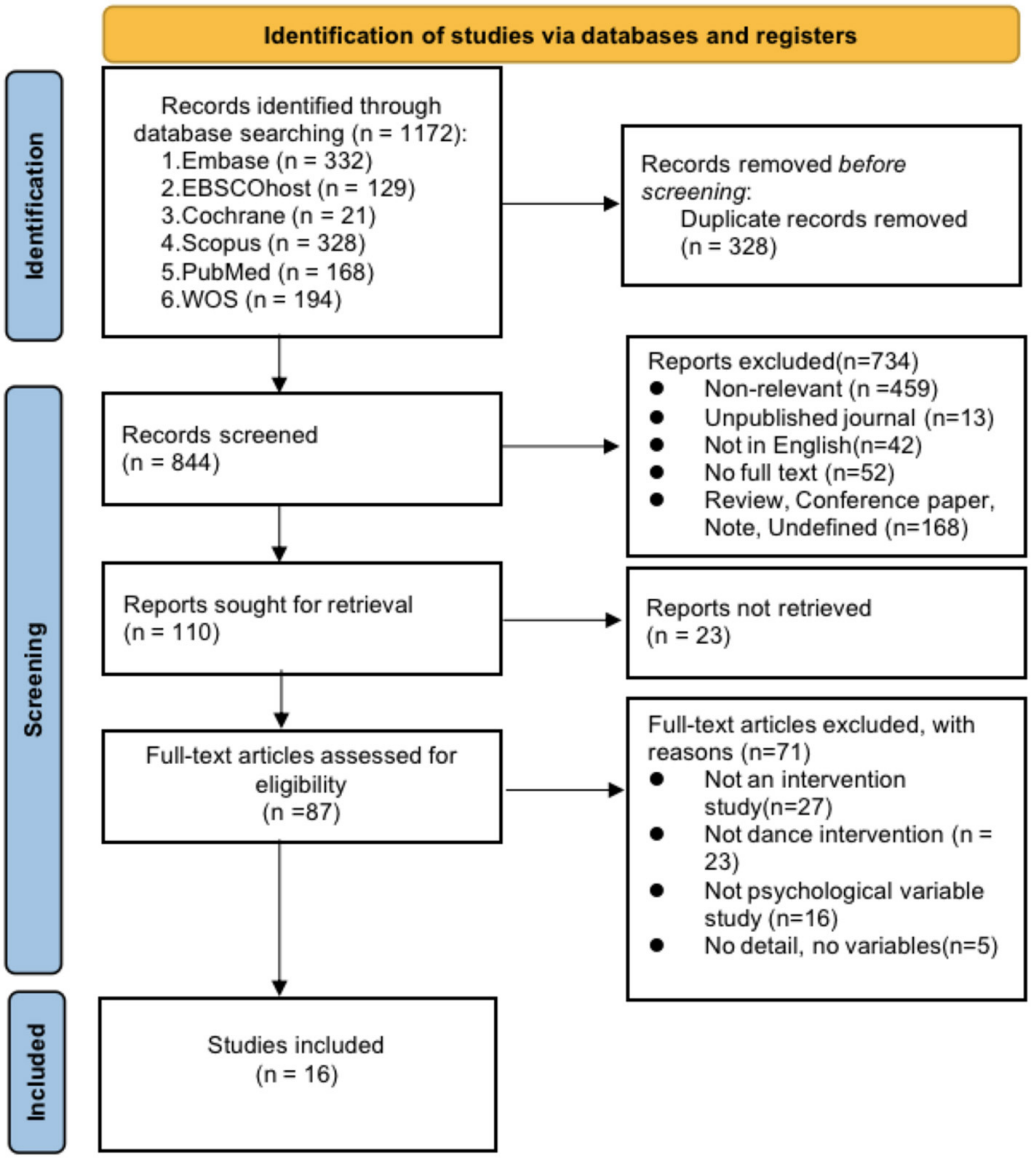
Preferred reporting items for systematic reviews and meta-analysis (PRISMA) flow chart of selection procedure.

A total of 1172 records were retrieved from Embase, EBSCOhost, Cochrane, Scopus, PubMed, and Web of Science. After removing duplicates using EndNote 20 citation management software, titles and abstracts were screened by two independent authors. Any disagreements were resolved through discussion with a third author. Following full-text review, 16 studies met the inclusion criteria. The study selection process is illustrated in Figure 1 according to the PRISMA guideline.

### Study quality assessment

Table 3 provides detailed information about PEDro scale scores in each study. Data from all studies had a score of 3 to 6 on the PEDro scale. This indicates that the inclusion quality is average. All studies specified their eligibility criteria and point estimates and variability. And all studies reported on between group comparisons except two studies (Domene et al., 2016b; Donath et al., 2014). None of the studies reported on blind subject, blind therapists, blind assessors and only one study (Barene et al., 2014) reported on concealed allocation. In the context of dance interventions, blinding is often not feasible due to the interactive and participatory nature of the activity. As a result, PEDro scores may not fully capture the methodological strengths of these studies. Therefore, rather than focusing solely on blinding, future studies should enhance other areas such as randomization procedures, outcome reporting, and intervention transparency to strengthen the overall evidence base for dance and health research.

**Table 3:** Summary of methodological quality assessment scores.

**References**	**Eligibility criteria**	**Random allocation**	**Concealed allocation**	**Baseline comparability**	**Blind subjects**	**Blind therapists**	**Blind assessors**	**Adequate follow-up**	**Intention to treat analysis**	**Between group comparisons**	**Point estimates and variability**	**PEDro score**
Domene et al. (2016b)	1	1	0	1	0	0	0	1	0	0	1	4
Barene et al. (2014)	1	1	1	1	0	0	0	0	1	1	1	6
Donath et al. (2014)	1	1	0	1	0	0	0	0	0	0	1	3
Norouzi et al. (2020)	1	1	0	1	0	0	0	1	1	1	1	6
Delextrat et al. (2016)	1	1	0	1	0	0	0	0	0	1	1	4
Pacheco et al. (2016)	1	0	0	1	0	0	0	0	0	1	1	3
Byrka and Ryczko (2018)	1	0	0	1	0	0	0	0	0	1	1	3
Rao et al. (2020)	1	0	0	1	0	0	0	0	0	1	1	3
Muyor et al. (2017)	1	0	0	1	0	0	0	0	0	1	1	3
Adilogullari (2017)	1	0	0	1	0	0	0	0	0	1	1	3
Marquez et al. (2017)	1	0	0	1	0	0	0	0	0	1	1	3
Meric and Ilhan (2016)	1	0	0	1	0	0	0	0	0	1	1	3
Barnet-Lopez et al. (2016)	1	1	0	0	0	0	0	0	0	1	1	3
Meekums et al. (2012)	1	1	0	0	0	0	0	0	0	1	1	3
Salmons et al. (2022)	1	1	0	0	0	0	0	0	0	1	1	3
Boing et al. (2023)	1	1	0	1	0	0	0	0	0	1	1	4

### Participant characteristics

The systematic review of 16 papers is summarized in Table 4, focusing on the following aspects: (1) Sample size: The number of participants ranged from 20 (Domene et al., 2016b) to 107 (Barene et al., 2014), with an average sample size of approximately 59 participants. (2) Gender: Eight articles investigated women-only samples (Barene et al., 2014; Boing et al., 2023; Delextrat et al., 2016; Domene et al., 2016b; Donath et al., 2014; Meekums et al., 2012; Norouzi et al., 2020; Pacheco et al., 2016), while the other eight included participants of both genders (Adilogullari, 2017; Barnet-Lopez et al., 2016; Byrka and Ryczko, 2018; Marquez et al., 2017; Meric and Ilhan, 2016; Muyor et al., 2017; Rao et al., 2020; Salmons et al., 2022). (3) Age: Nine studies were conducted on adults aged 18 and above (Barene et al., 2014; Barnet-Lopez et al., 2016; Byrka and Ryczko, 2018; Delextrat et al., 2016; Domene et al., 2016b; Meekums et al., 2012; Muyor et al., 2017; Norouzi et al., 2020; Rao et al., 2020), three on college students aged 18-24 (Adilogullari, 2017; Donath et al., 2014; Meric and Ilhan, 2016), and four on older adults aged 50 and above (Boing et al., 2023; Marquez et al., 2017; Pacheco et al., 2016; Salmons et al., 2022).

**Table 4:** Characteristics of the studies examined in the present review.

**References**	**Type of sample**		**Detail of intervention**	**Type of dance**	**Instruments**	**Type of research design**	**Outcomes**
	**Populations**	**Age**					
Domene et al. (2016b)	*N* = 20; Adult female	18–64	Fre:2 times/week Duration:8 weeks	Zumba	HRQoL	Pre-post test	Physical appearance↑ mental health ↑;
Barene et al. (2014)	*N* = 107; Female hospital employees	25–65	Fre:2–3 times/week Duration:12 weeks	Zumba	Heart rate measurements; VAS	Pre-post test	Bodily attractiveness ↑
Donath et al. (2014)	*N* = 30; University female students	18–24	Fre:2 times/week Duration:8 weeks	Zumba	QoL	Pre-post test	Self-performance↑
Norouzi et al. (2020)	*N* = 60; Adult female	30–40	Fre:3 times/week Duration:12 weeks	Zumba	Depressive symptoms	Pre-post test	Negative Evaluation↓
Delextrat et al. (2016)	*N* = 22 Adult female	20–32	Fre:3 times/week Duration:8 weeks	Zumba	PSPP	Pre-post test	Self-performance↑; Physical self-esteem ↑
Pacheco et al. (2016)	*N* = 27; Older female	60–76	Fre:3 times/week Duration:12 weeks	Folk dances	HRQoL	Pre-post test	Physical fitness ↑; physical appearance↑
Byrka and Ryczko (2018)	*N* = 64; Adult	18–63	Fre:2 times/week Duration:5 weeks	Salsa	MVPA	Pre-post test	Physical appearance↑; Self-esteem ↑
Rao et al. (2020)	*N* = 80; Adult	30–50	Fre:3 times/week Duration:8 weeks	Aerobic dance	WHO QOL scale	Pre-post test	Anxiety ↓; Self-esteem ↑; Quality of life ↑
Muyor et al. (2017)	*N* = 40; Adult	20–25	Fre:3 times/week Duration:6 weeks	Latin	Spinal morphology	Pre-post test	Body posture↑; Physical appearance↑; Self-esteem ↑
Adilogullari (2017)	*N* = 60; University students	18–23	Fre:2 times/week Duration:12 weeks	Latin	SPAI	Pre-post test	Social physique anxiety ↓
Marquez et al. (2017)	*N* = 57; Older adults	≥ 55	Fre:1 times/week Duration:12 weeks	Latin	Self-Confidence Scale	Pre-post test	Self-confidence ↑
Meric and Ilhan (2016)	*N* = 60; University students	18–23	Fre:1 times/week Duration:12 weeks	Latin	Self-Confidence Scale	Pre-post test	Self-confidence ↑
Barnet-Lopez et al. (2016)	*N* = 42; Adult	30–60	Fre:2 times/week Duration:12 weeks	DMT	Koppitz human figure drawing test	Pre-post test	Physical self-esteem ↑
Meekums et al. (2012)	*N* = 92; Adult female	20–50	Fre:2 times/week Duration:5 weeks	DMT	CORE-OM; SIBID; self-esteem scale	Pre-post test	Psychological↓; body image↓; self-esteem ↑
Salmons et al. (2022)	*N* = 49; old adult	≥50	Fre:1 times/week Duration:12 weeks	DMT	Beck's depression inventory; hospital anxiety and depression scale; self-esteem scale	Pre-post test	Anxiety ↓; Self-esteem ↑
Boing et al. (2023)	*N* = 74 breast cancer survivors	52–58	Fre:3 times/week Duration:16 weeks	Belly dance	Body image after breast cancer questionnaire; Rosenberg self-esteem scale; female sexual function index	Pre-post test	Body image ↑; Self - esteem ↑

HRQoL, health-related quality of life; VAS, visual analog scales; MVPA, moderate and vigorous physical activity; BAQ, body attitude questionnaire; PSPP, physical self-perception profile; WHO QOL scale, World Health Organization Quality Of Life Scale; SPAI, social physique anxiety inventory; DMT, dance movement therapy; DEBQ, Dutch eating behavior questionnaire; CORE-OM, clinical outcomes in routine evaluation – outcome measure; SIBID, situational inventory of body image Dysphoria; Higher, ↑; Lower, ↓.

### Dance intervention characteristics

In this review, “Latin dance” refers to ballroom Latin styles included in the studies reviewed, such as Cha Cha, Rumba, Jive, and Samba, which are distinct from Latin social dances like Salsa. The dance interventions examined in the included studies varied in type, duration, and frequency. A range of dance styles were employed, including Zumba (*n* = 5), Latin dance (*n* = 4), Dance Movement Therapy (*n* = 3), Salsa (*n* = 1), Colombian Caribbean Folk dances (*n* = 1), Aerobic dance (*n* = 1), and Belly dance (*n* = 1). The duration of the interventions ranged from 5 to 16 weeks, with 12-week programs being the most common. In terms of frequency, most interventions were conducted two to three times per week, while a few studies implemented once-a-week sessions.

### Outcome

Across the 16 included studies, the majority reported that dance interventions were associated with improvements in either perceived physical appearance or PSE. Thirteen studies showed enhancements in PSE, often through positive changes in perceived body image and bodily self-perception (Barene et al., 2014; Barnet-Lopez et al., 2016; Boing et al., 2023; Byrka and Ryczko, 2018; Delextrat et al., 2016; Domene et al., 2016b; Marquez et al., 2017; Meekums et al., 2012; Meric and Ilhan, 2016; Muyor et al., 2017; Pacheco et al., 2016; Rao et al., 2020; Salmons et al., 2022). Five studies found that dance also reduced negative self-evaluation and SPA, particularly through increased self-performance and confidence (Adilogullari, 2017; Donath et al., 2014; Norouzi et al., 2020; Rao et al., 2020; Salmons et al., 2022). Two studies reported simultaneous improvements in both outcomes (Rao et al., 2020; Salmons et al., 2022).

Among the dance styles included in the studies, Zumba and Latin dance were the most frequently studied. Latin dance was shown to reduce SPA while enhancing PSE and self-confidence (Adilogullari, 2017; Marquez et al., 2017; Meric and Ilhan, 2016; Muyor et al., 2017). Zumba, DMT, and Belly dance were associated with improvements in body image and reductions in anxiety (Barene et al., 2014; Barnet-Lopez et al., 2016; Boing et al., 2023; Delextrat et al., 2016; Domene et al., 2016a; Donath et al., 2014; Meekums et al., 2012; Norouzi et al., 2020; Salmons et al., 2022). Perceived physical appearance and bodily attractiveness were assessed through self-report measures and are considered subdimensions of PSE rather than separate outcome variables.

## Discussion

This review provides comprehensive evidence that dance interventions can positively impact SPA and PSE across a wide range of populations. For example, Zumba and Aerobic dance were linked to decreased SPA in women aged 18 to 40 (Donath et al., 2014; Norouzi et al., 2020), and DMT was effective for older adults, showing improvements in anxiety reduction and PSE (Salmons et al., 2022). Gender-specific outcomes were also evident: women demonstrated greater improvements in SPA reduction than men, likely due to higher initial levels of body-related anxiety (Adilogullari, 2017). These findings suggest that dance, as both a physical and expressive activity, may serve as a promising non-pharmacological approach to improving mental wellbeing. The psychological benefits were especially pronounced in women and older adults, with variations depending on dance type, frequency, and program duration (Abdollahi et al., 2023; Zhang et al., 2023).

Dance has been shown to reduce SPA by improving body confidence and reducing negative evaluation from others. This may be attributed to several mechanisms: social bonding during group sessions, redirection of attention from body image to movement, and the mastery of dance skills enhancing self-efficacy. These findings align with recent systematic reviews (Liu et al., 2023; Tomaszewski et al., 2023; Wang et al., 2022), which confirm the potential for dance to promote positive psychological states.

Dance also improved PSE across demographics. Studies found that Latin and Salsa dance improved self-perception and confidence in college students (Barnet-Lopez et al., 2016; Byrka and Ryczko, 2018; Meric and Ilhan, 2016), while DMT and Belly dance enhanced self-esteem and body image in older adults and breast cancer survivors (Boing et al., 2023; Meekums et al., 2012). These improvements are thought to arise from internal shifts in self-perception rather than external appearance, promoting a holistic appreciation of the body (Haugen et al., 2013; Tomaszewski et al., 2023).

Recent reviews (Dale et al., 2019; Tomaszewski et al., 2023; Zhou et al., 2023) support these outcomes and highlight how different dance forms and intervention durations may impact age and gender groups differently. Notably, more intense and longer programs appeared to yield stronger improvements.

In conclusion, dance interventions appear to positively influence both SPA and enhance PSE. While consistent trends are evident, future research should expand the demographic scope—particularly including adolescents and underrepresented genders—and explore the long-term effects and mechanisms of dance-based interventions in promoting mental wellbeing.

## Limitations and future research

While this study contributes valuable insights into the effects of dance on SPA and PSE, it is essential to acknowledge the identified limitations. Addressing these limitations through future research endeavors would strengthen the robustness and applicability of the findings.

1. The study focused on adult participants and college students, with no studies assessing adolescents. Consequently, the findings may not fully capture the effects of dance on the mental health of younger populations. Future research should strive to include adolescents and children to provide a comprehensive understanding of the impact of dance on different age groups.
2. The literature reviewed primarily compared dance interventions with traditional exercise or daily activities. However, there was a lack of comparison between dance and other innovative forms of exercise such as high-intensity interval training (HIIT), mind-body practices like yoga and pilates, or alternative movement therapies. Additionally, there was insufficient exploration of the relative effectiveness of different dance styles.
3. The inclusion of different dance styles and age groups prevented the performance of a meta-analysis, which is generally preferred over a systematic review when feasible.
4. Gender bias emerged as a concern, as eight out of the 16 studies exclusively represented female. This exclusive representation limits the generalizability of the findings to a broader population. While future research should strive for more balanced gender representation, it is important to note that this may be difficult to address given that fewer men tend to participate in dance-based activities. nonetheless, efforts should be made to ensure a more comprehensive understanding of the effects of dance interventions across diverse populations.

## Conclusion

In conclusion, this review demonstrates that dance interventions can effectively improve SPA and enhance PSE. Dance styles such as Zumba, Latin dance, Salsa, DMT, Colombian Caribbean Folk dances, Aerobic dance, and Belly dance have shown positive impacts on these psychological aspects compared to control groups, irrespective of age. Moreover, the results suggest that dance may have a more pronounced effect on improving PSE and reducing SPA in women compared to men. However, it is essential to acknowledge potential study bias and limitations. Overall, incorporating dance into mental health interventions holds promise for fostering wellbeing and body image perception among diverse populations.

## Data Availability

The datasets presented in this study can be found in online repositories. The names of the repository/repositories and accession number(s) can be found in the article/supplementary material.
